# Prognostic analysis of bladder cancer with neddylation-related genes

**DOI:** 10.1186/s41065-025-00463-y

**Published:** 2025-06-16

**Authors:** Huang Xiaolong, Deng Min, Zhang Sizhou, Hu Daorong, Pan Juncheng, Wang Yanjian, Li Jie Gu Hong, Zheng Ji, Wu Qingjian

**Affiliations:** 1 Department of Urology, the Second Affiliated Hospital, Third Military Medical University (Army Medical University), Xinqiao Road No.83, Chongqing, 400037, P. R. China; 2 Department of Urology, People’s Hospital of Chongqing Hechuan, Chongqing, 401520 PR China

**Keywords:** Bladder cancer, Neddylation, Clinical prognostic analysis, Molecular biomarkers, Personalized medicine

## Abstract

**Background:**

Previous studies have demonstrated a close association between neddylation modifications and tumor progression as well as alterations in the microenvironment. This study aimed to explore the role of neddylation in bladder cancer (BLCA) progression and its prognostic significance.

**Methods:**

Gene expression data from the TCGA database were analyzed to identify neddylation gene modules using the limma software package and weighted gene co-expression network analysis (WGCNA). Prognostic models based on neddylation-related genes were subsequently developed through LASSO and Cox regression analyses. Additionally, a protein–protein interaction (PPI) network, gene set enrichment analysis (GSEA), and BLCA single-cell sequencing data were utilized to explore the functional roles of hub genes in BLCA and their impact on biological pathways. The expression of these hub genes was further validated in clinical samples via RT-qPCR.

**Results:**

WGCNA analysis revealed 1412 neddylation-related hub genes. LASSO and Cox regression analyses subsequently identified six key genes: CUL1, PUM2, UBE2D3, HIF3A, COPS2, and DDB1. Transcriptomic data and RT-qPCR findings indicated that PUM2 and HIF3A exhibited high expression levels in normal tissues, while DDB1 showed increased expression in tumor tissues; no significant changes were observed for CUL1, COPS2, and UBE2D3. By integrating these gene expressions with significant clinical features, a prognostic model was constructed that demonstrated excellent diagnostic efficiency (AUC: 0.793 at 1 year, 0.792 at 3 years, and 0.773 at 5 years). In addition, single cell sequencing highlighted the potential role of these genes in modulating immune responses and mediating interactions between tumor cells and immune cells. GSEA also suggested that DDB1 may play a crucial role in orchestrating key biological processes associated with BLCA, particularly in activating apoptotic signaling pathways.

**Conclusion:**

The six neddylation-related genes (CUL1, PUM2, UBE2D3, HIF3A, COPS2, and DDB1) emerge as potential independent indicators of survival in patients with BLCA, and the constructed survival models exhibit significant diagnostic efficacy.

**Supplementary Information:**

The online version contains supplementary material available at 10.1186/s41065-025-00463-y.

## Introduction

Bladder cancer (BLCA) represents the most prevalent malignant tumor of the urinary tract, exhibiting an incidence rate 3–4 times higher in men and a mortality rate exceeding twice that in women [1–3]. The disease is broadly classified into muscle-invasive BLCA (MIBC) and non-muscle-invasive BLCA (NMIBC), with approximately 75% of patients receiving an initial diagnosis of NMIBC [4, 5]. Nearly 70% of NMIBC cases recur within five years, while 10–20% advance to either highly invasive or distant metastatic stages [6, 7]. Evidence supports the notion that BLCA encompasses a group of molecularly heterogeneous disorders, each following distinct clinical courses and responding variably to treatment; its development involves multiple molecular pathways [8, 9]. Furthermore, recent investigations have highlighted a significant correlation between neddylation modifications and both tumor progression and microenvironmental alterations [10, 11]. Despite these findings, the impact of neddylation-related genes and their modifications on the progression and prognosis of BLCA remains poorly understood.

Studies indicate that enzymes involved in neddylation modifications occur at elevated levels in tumors compared to adjacent normal tissues, sparking considerable interest in the link between neddylation and tumor biology [8–11]. Neddylation plays a role in several cellular processes, including cell cycle regulation, apoptosis, senescence, autophagy, angiogenesis, and modulation of the tumor immune response [10–13]. Disruption of this modification process has been implicated in cancer development, as abnormal neddylation can drive uncontrolled cell proliferation, tumor expansion, and metastasis [12, 13]. Furthermore, overexpression and activation of key neddylation enzymes, namely, the E1 NEDD8 activating enzyme (NAE), E2 NEDD8 conjugating enzyme, and E3 neddylation ligase, are frequently observed in lung, pancreatic, and bladder cancers [14].This overexpression is often correlated with poorer prognosis and reduced survival, highlighting the significant impact of neddylation on cancer progression [15]. Consequently, targeting neddylation has emerged as a promising strategy for cancer therapy. The present investigation systematically identified pivotal gene targets and clinical prognostic markers related to neddylation that influence BLCA outcomes, utilizing bioinformatic analyses and clinical specimen validation to provide enhanced insights into BLCA management and prognostic evaluation.

## Materials and methods

### Neddylation-related gene screening and differential gene expression analysis in BLCA

Differential expression analysis of gene expression data from BLCA patients in The Cancer Genome Atlas (TCGA) was conducted using the R language limma package. Genes meeting the criteria of P < 0.05 and|LogFC| > 1 were designated as differentially expressed. The analysis results were visualised using volcano plots generated by the ‘ggplot’ software package [16]. A total of 246 neddylation-related genes were retrieved from the KEGG database [17] and compared with the BLCA differential genes to identify potential overlap.

### WGCNA and PPI networks analysis

Weighted gene co-expression network analysis (WGCNA) was applied to BLCA-associated target genes using the R package WGCNA [18]. This approach facilitated the construction of weighted gene co-expression networks and enabled the identification of clusters of genes associated with bladder cancer. Subsequently, a PPI network was constructed from the genes identified via WGCNA by leveraging the STRING website [19]. The resulting network was rendered using Cytoscape 3.9.1 software (Cytoscape Consortium, USA) for visualization purposes [20].

### Construction of a prediction model

The R package glmnet facilitated LASSO Cox regression analyses on the selected key genes to pinpoint the most robust prognostic markers [21]. Subsequent refinement involved single-gene Cox regression to further screen these key genes. Finally, multiple Cox regression analyses allowed the development of a risk score formula that calculates the probability of poor survival for each sample by applying coefficient weighted expression values of the prognostic genes [22].

### Nomogram prediction model creation and analysis

Lasso regression analysis was utilized to pinpoint optimal prognostic factors, drawing on clinical parameters including age, gender, and lymphatic infiltration as a foundation for the evaluation [23]. Univariate and multivariate COX analyses were subsequently employed to identify independent prognostic indicators. These factors were then integrated into line plots to facilitate the assessment of overall survival (OS).

### Survival analysis and single-cell sequencing analysis

The Kaplan-Meier method was used to estimate overall survival (OS) probabilities, while the log-rank test was used to assess the significance of differences in OS between groups, with p-values < 0.05 considered statistically significant [24]. To gain a comprehensive understanding of the expression of the neddylation-related gene in bladder cancer cells, single-cell RNA sequencing data (GSE135337) was obtained from the GEO database for external validation. Analysis was performed using the Seurat software package in R (version 3.1.1). Resulting Seurat objects underwent rigorous data quality control and normalization, screening for highly variable genes, cellular downscaling clustering, and identification of differentially expressed genes among clusters [25]. Subsequently, cell types were annotated based on the raw text annotation method.

### Gene set enrichment analysis (GSEA)

GSEA serves to identify gene sets associated with specific phenotypes by means of comparative analysis. Prognostic genes, as determined by Cox regression and stratified by median values, underwent comparison with the C2.CP.KEGG.v7.4 and C5.GO.v7.4 gene sets. The clusterProfiler package in R facilitated this analysis, aiming to clarify the role of these genes in bladder cancer. Gene sets displaying a P-value below 0.05 and an FDR q-value under 0.05 were deemed significant, highlighting enriched pathways that may be pivotal in understanding the disease mechanisms.

### Quantitative real-time polymerase chain reaction analysis

Cancer tissues and corresponding adjacent tissues from 12 BLCA patients were obtained at People’s Hospital of Hechuan District for qRT-PCR analysis. Approval for the study was granted by the Committee of People’s Hospital of Hechuan District, and all procedures adhered to the Declaration of Helsinki. The qRT-PCR protocols followed methods previously detailed in the literature [26]. The PCR primer sequences are provided below:

- GAPDH, forward: 5′-TCGGAGTCAACGGATTTGGT-3′;
- GAPDH, reverse: 5′-TTCCCGTTCTCAGCCTTGAC-3′;
- CUL1, forward: 5′-ACGTGGAATTGGGGCTGAAT-3′;
- CUL1, reverse: 5′-ACGAGCCTCTGCCTTTTTCA-3′;
- PUM2, forward: 5′-TGGCAGCCATGTTGTTGTTT-3′;
- PUM2, reverse: 5′-CTCTCTCCATTCATCATCCCCA-3′;
- UBE2D3, forward: 5′-AAGAAGGTGCTGTTCCGAGA-3′;
- UBE2D3, reverse: 5′-ACACAGGCGCCTCTTCAC-3′;
- HIF3A, forward: 5′-GCAGCGCGCAAGGTCG-3′;
- HIF3A, reverse: 5′-GCAGGTAGCTGATGGTGAGG-3′;
- COPS2, forward: 5′-AGTCGTGCCAGACTGATGA-3′;
- COPS2, reverse: 5′-GAGGGATGGCAGACTTGATGT-3′;
- DDB1, forward: 5′-GCCACCTGTCTTTTCGCTTG-3′;
- DDB1, reverse: 5′-GCCGAAGTAAAGTGTCCGGT-3′;

The 2-ΔΔCt method was conducted to calculate the RNA expression. Student’s t-test was used to compare the expression level of each RNA between different groups.

### Statistical analysis

All statistical analyses were carried out using the R programming language. Data comparisons across groups were performed with either an independent-samples t-test or a Kruskal test. A p-value below 0.05 was deemed statistically significant.

## Results

### Screening for BLCA differentiation genes

**Fig. 1:**
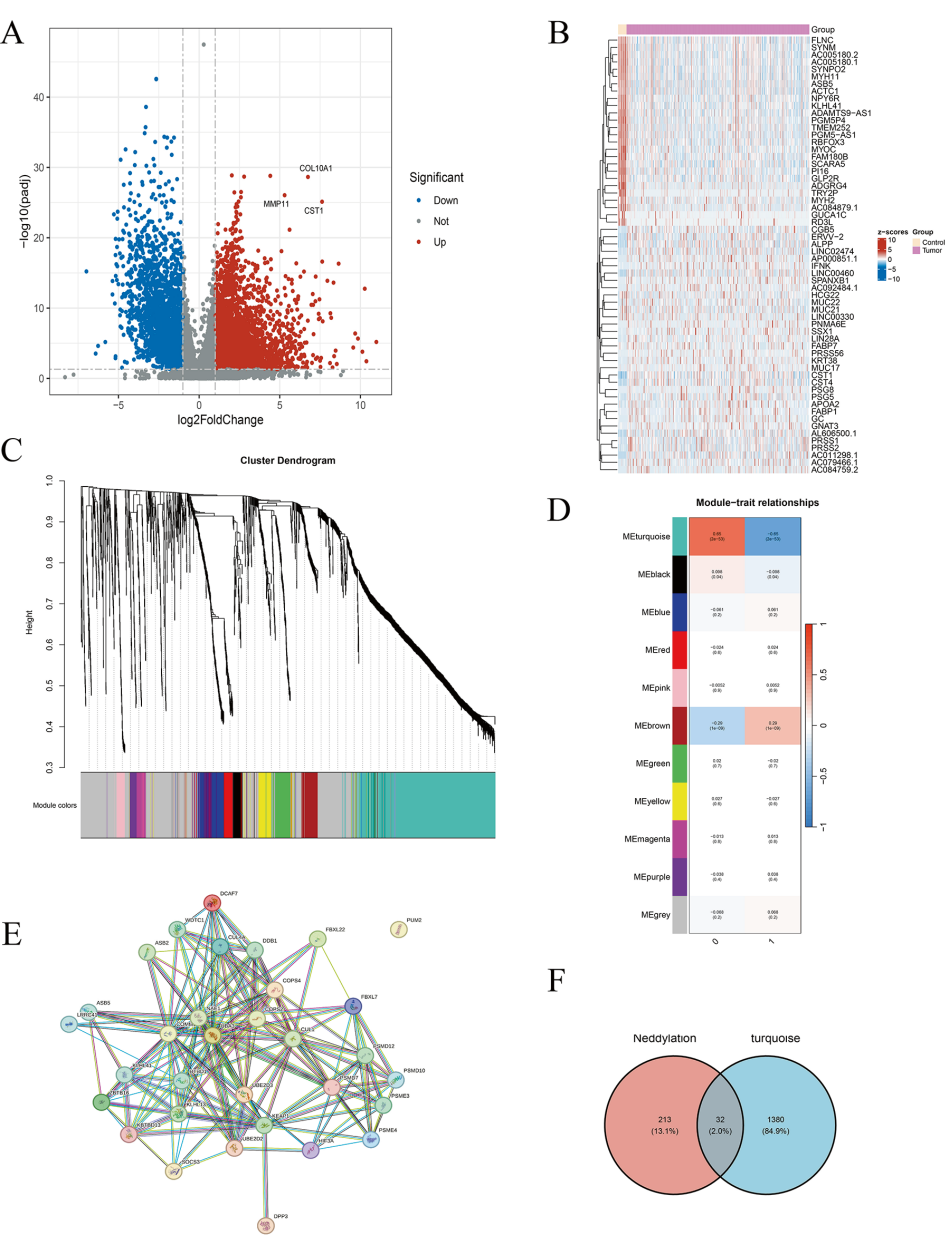
Differentially expressed genes between BLCA tissues and normal tissues. (**A**) Volcano plot. (**B**) Heatmap of differentially expressed genes in BLCA: blue indicates downregulation, red indicates upregulation. (**C**) Identification of BLCA gene modules in the TCGA dataset using WGCNA. (**D**) Relationship between modules and traits. (**E**) Protein-protein interaction (PPI) network construction. (**F**) Intersection results of hub genes between BLCA-associated genes and neddylation genes

Figure 1A displays a heat map that illustrates the identification of differential genes between normal and BLCA tissues. The analysis pinpointed 2974 genes exhibiting significant expression differences, marking them as potential targets related to BLCA. Following this, the expression patterns of these differential genes in the two groups were visualized in another heat map (Fig. 1B). Each row represents a gene and each column a sample; darker shades indicate lower expression and lighter shades indicate higher expression.

As shown in Fig. 1C, WGCNA classified these 2974 differential genes associated with prognosis into 12 distinct modules. A detailed examination of the relationships between these modules and BLCA traits identified 1412 genes within the turquoise module. An intersection with neddylation-related genes further refined the list, ultimately extracting 32 neddylation-associated genes (Fig. 1E). The protein interaction network for these genes is presented in Fig. 1D.

### Univariate and multivariate Cox regression analysis

**Fig. 2:**
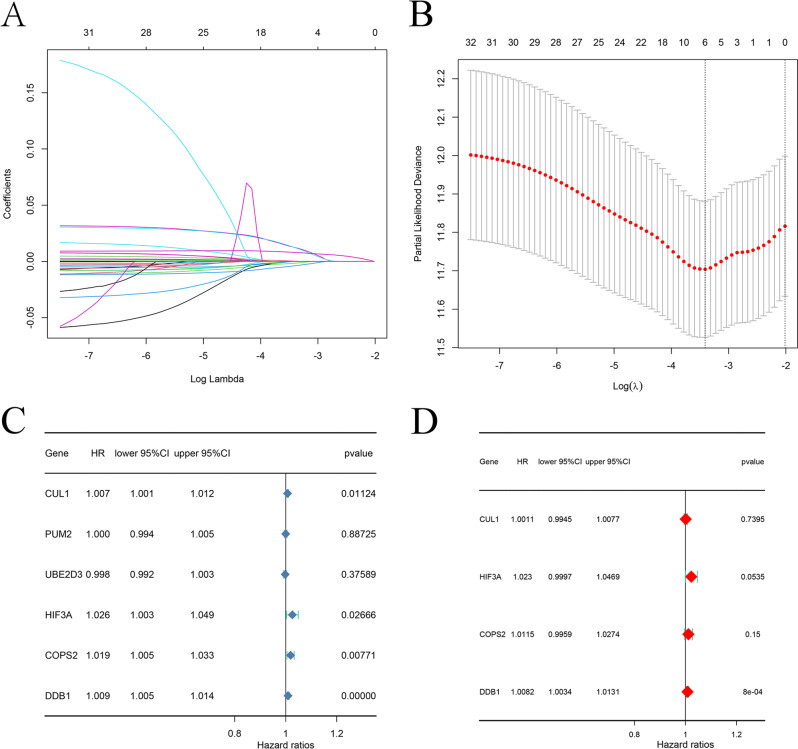
Univariate and multivariate Cox regression analyses. (**A** and **B**) LASSO regression analysis of the time of minimised model error. log(λ) = -3.436 and 6 genes are selected for further survival analysis. (**C**) Univariate Cox regression analysis. (**D**) Multivariate Cox regression analysis

**Fig. 3:**
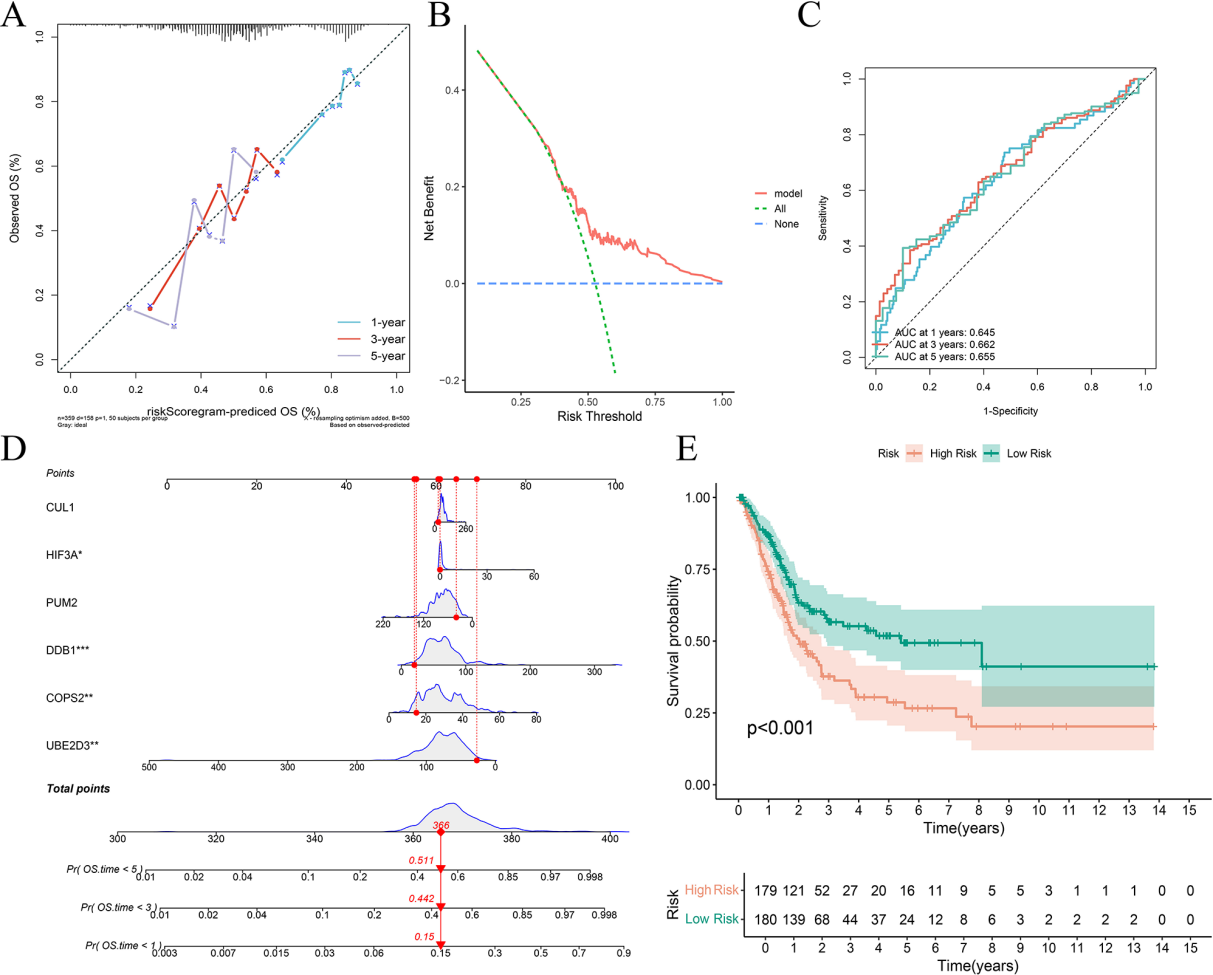
Receiver Operating Characteristic (ROC) and Survival Analysis. (**A**) Calibration curve of multivariate Cox regression analysis. (**B**) Decision curve analysis (DCA) curve of multivariate Cox regression analysis. (**C**) Receiver operating characteristic (ROC) curve of multivariate Cox regression analysis. (**D**) Nomogram plot using risk scores from CUL1, PUM2, UBE2D3, HIF3A, COPS2 and DDB1. (**E**) Multivariate Cox regression risk survival analysis

Subsequent lasso regression analysis on the 32 genes yielded eight critical candidates with potential prognostic significance in BLCA patients (Fig. 2A and B). Univariate and multivariate Cox regression analyses were performed to evaluate whether risk scores derived from six key genes, namely CUL1, PUM2, UBE2D3, HIF3A, COPS2, and DDB1, could serve as independent prognostic predictors of BLCA beyond traditional clinical parameters. Both analyses identified DDB1 as an independent prognostic factor for bladder cancer (Fig. 2C-D), with hazard ratios of 1.009 (95% CI 1.005–1.014) and 1.0082 (95% CI 1.0034–1.0131), respectively, indicating statistical significance. Further multivariate Cox regression confirmed that HIF3A, DDB1, COPS2, and UBE2D3 may independently indicate bladder cancer prognosis (Fig. 3A-D). A subsequent hazard survival analysis reinforced these findings by demonstrating a significantly lower overall survival rate in the high-risk group compared to the low-risk group (Fig. 3E).

### Differential and prognostic analysis

**Fig. 4:**
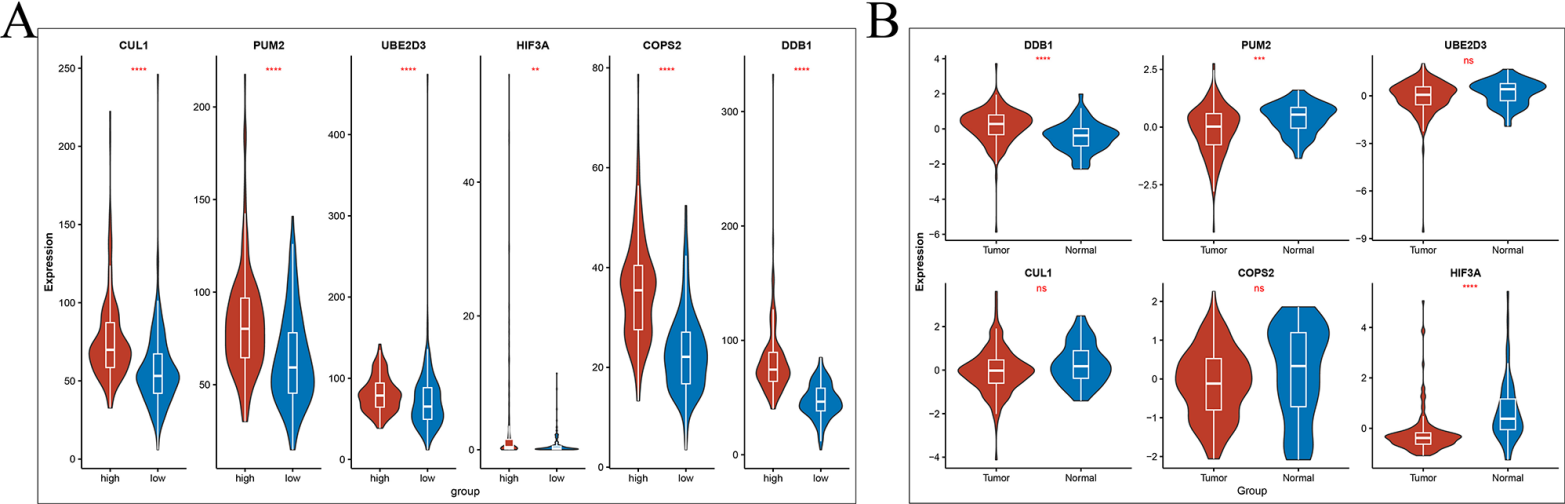
Differential gene expression expression. (**A**) Gene expression in high and low risk groups. (**B**) Hub gene expression between tumour and normal samples in transcriptome data. (**C**) Hub gene expression between tumour and normal samples in clinical specimen

**Fig. 5:**
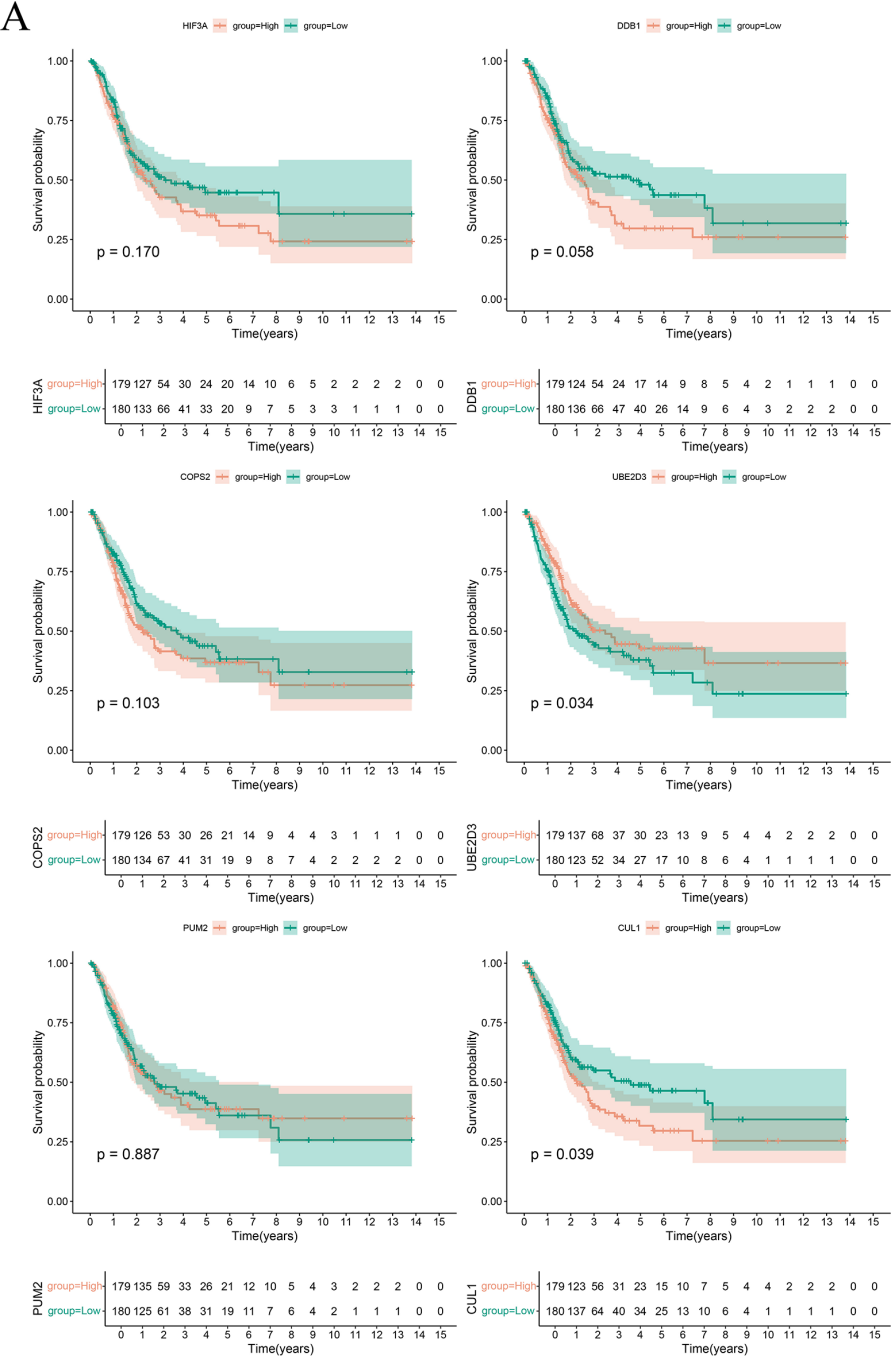
Prognostic survival analysis

A comprehensive investigation into differential gene expression between the high-risk and low-risk groups focused particularly on the expression levels of the prognostic genes. The analysis revealed that genes such as CUL1, PUM2, UBE2D3, COPS2 and DDB1 showed significantly increased expression levels in the high-risk group (Fig. 4A). Notably, transcriptomic data and RTqPCR results demonstrated that while PUM2 and HIF3A were highly expressed in normal tissues, DDB1 showed increased expression in tumor tissues, with CUL1, COPS2, and UBE2D3 remaining unchanged (Fig. 4B and C). In contrast, HIF3A was the only gene with a markedly reduced expression level in the high-risk group compared to the low-risk group. This distinct pattern shows the molecular heterogeneity between the groups and suggests that these genes could serve as valuable markers for assessing BLCA aggressiveness and guiding patient stratification. Furthermore, prognostic survival analysis for the six key genes (Fig. 5A) indicated that patients with elevated UBE2D3 expression tend to have a better survival prognosis, whereas those with higher CUL1 expression experience poorer outcomes.

### Nomogram prediction model creation and analysis

**Fig. 6:**
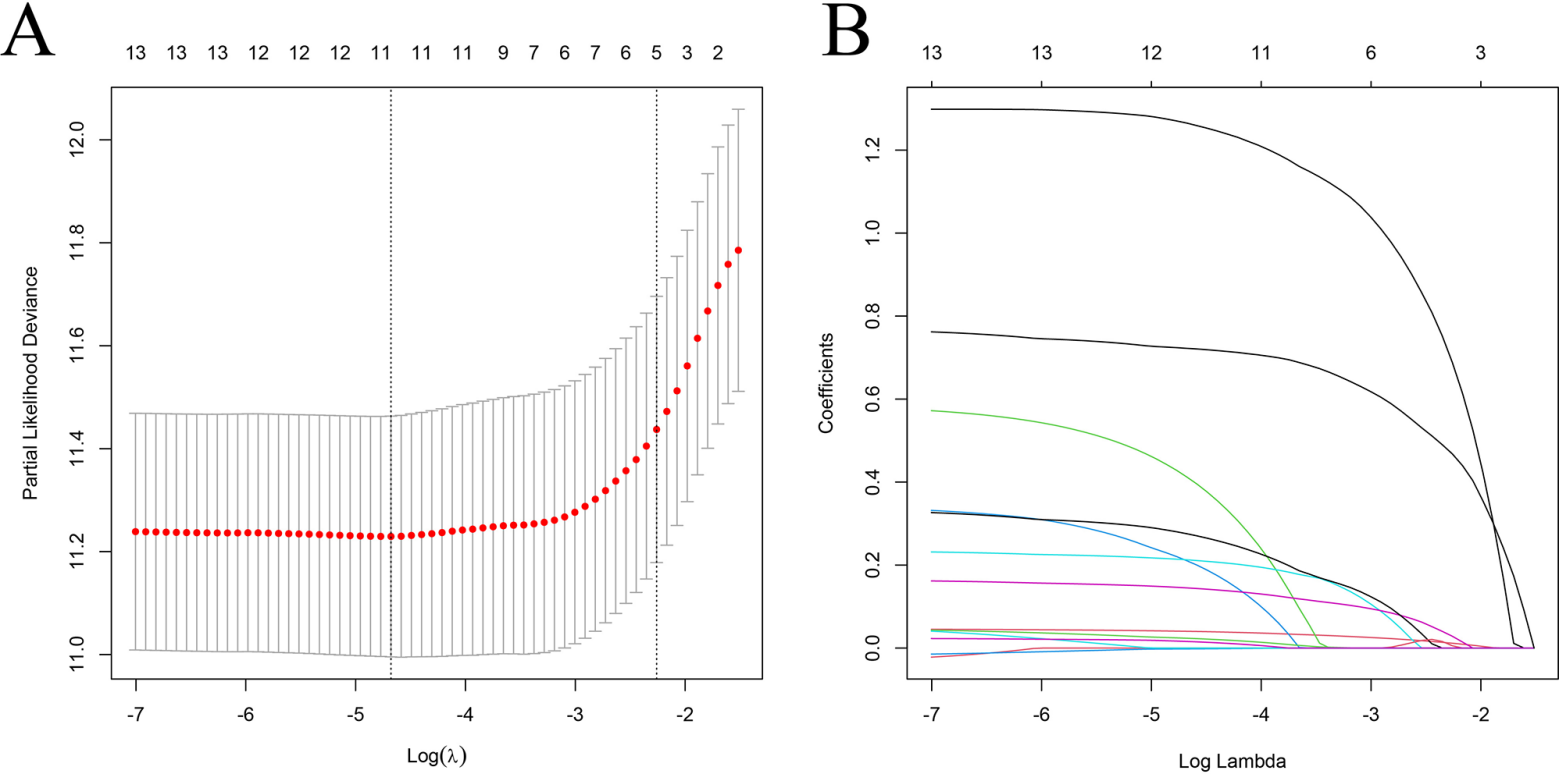
LASSO regression analysis. (**A**) Cross-validation identifies log(λ) = -2.31 as the optimal value minimizing model error. (**B**) At this threshold, four prognosis-related factors are selected for further survival analysis

**Fig. 7:**
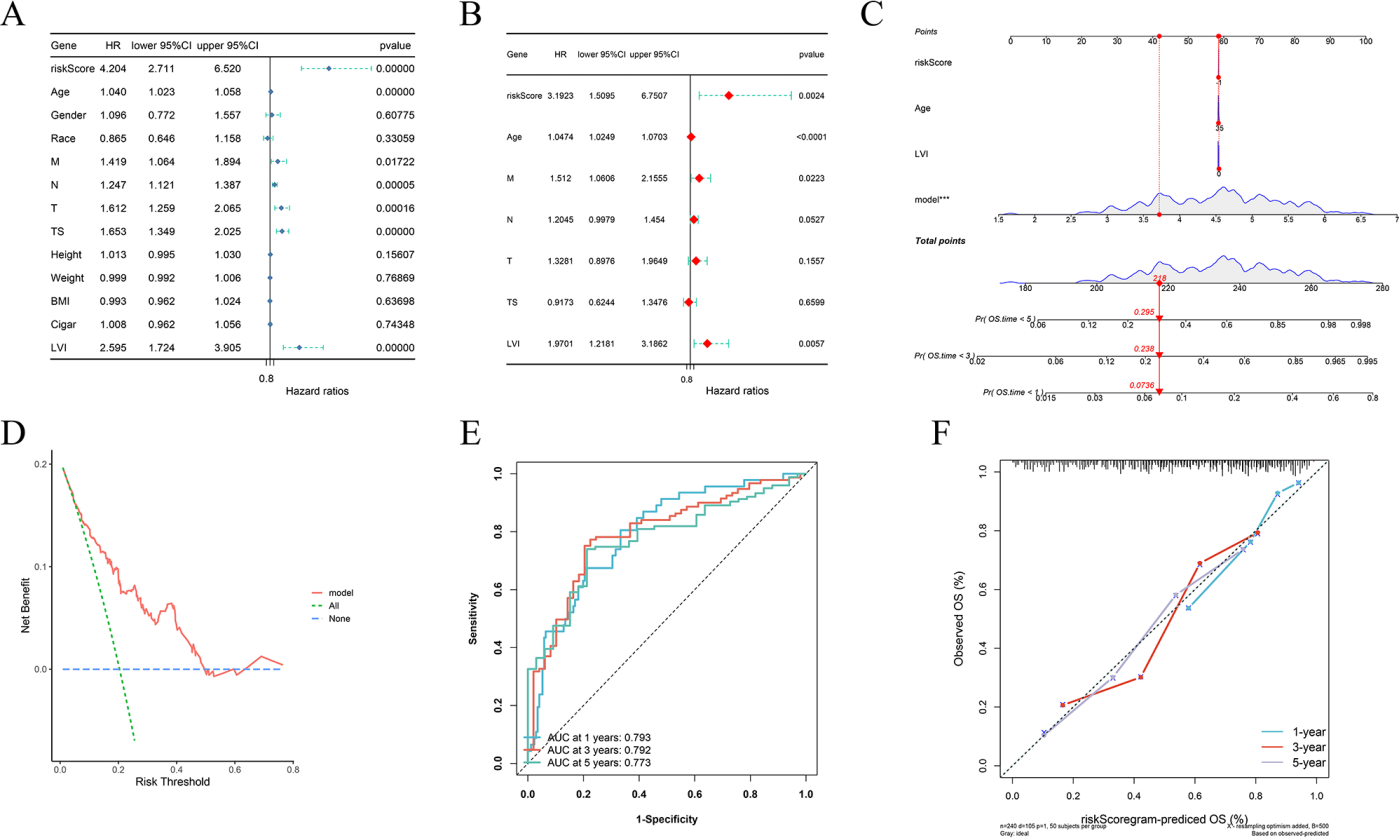
Nomogram prediction model creation and analysis. (**A**) Univariate Cox regression analysis. (**B**) Multivariate Cox regression analysis. (**C**) Nomogram plot using risk scores from riskscore, M, Age, and LVI. (**D**) Decision curve analysis (DCA) curve of multivariate Cox regression analysis. (**E**) Receiver operating characteristic (ROC) curve of multivariate Cox regression analysis.(**F**) Calibration curve of multivariate Cox regression analysis

A lasso regression analysis was employed to sift through various prognostic factors, resulting in the identification of three significant clinical parameters: age, metastasis (M), and nodal (N) status in conjunction with lymphovascular invasion (LVI) (Fig. 6A-B). Subsequent univariate Cox regression analysis confirmed that these factors serve as independent clinical predictors of BLCA (Fig. 7A). After constructing models based on the univariate findings, multivariate Cox regression using the survival package in R was applied to evaluate the independent contribution of risk scores in prognosis estimation (Fig. 7B-F). The analysis revealed that a new model, which integrates six previously identified genes with these clinical traits, can accurately predict the survival outcome of bladder cancer patients, achieving AUC values of 0.793 at 1 year, 0.792 at 3 years, and 0.773 at 5 years. The final model is defined by the equation: model = 1.186 × risk score + 0.046 × age + 0.5523 × M + 0.873 × LVI.

### Single-cell RNA sequencing analysis

**Fig. 8:**
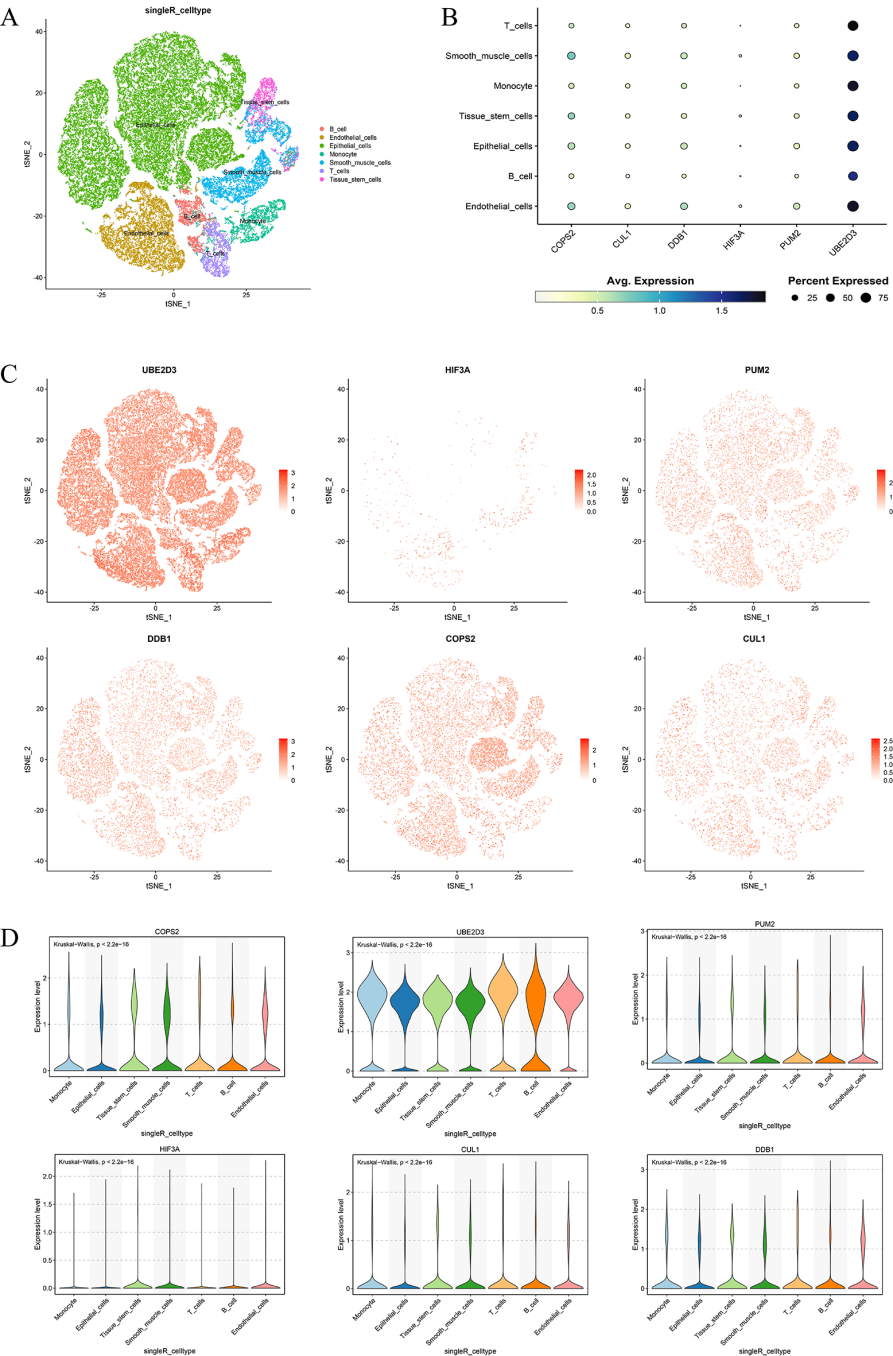
Single-cell RNA sequencing analysis. (**A**) 7 major immune cell clusters. (**B**) Dot plot of hub gene expression: dot size reflects the percentage of expressing cells, and color intensity indicates expression level. (**C**) UMAP plot of hub gene expression.(**D**) Violin diagram of hub genes

Single-cell RNA sequencing was carried out for further verification. A total of 52,721 cells were acquired from the GEO database and sorted into clusters, which resulted in the identification of seven distinct cell types (Fig. 8A). The expression levels of the genes CUL1, PUM2, UBE2D3, HIF3A, COPS2, and DDB1 were then analyzed across these seven clusters (Fig. 8B-D). UBE2D3 and COPS2 exhibited high expression in all cell types, whereas HIF3A and CUL1 showed consistently low expression. DDB1 showed increased expression in endothelial, epithelial and smooth muscle cells, and PUM2 was predominantly expressed in endothelial cells. The expression differences for these six genes within each cell type are further illustrated by violin plots (Fig. 8D).

### Analysis of key pathways associated with BLCA by DBB1

**Fig. 9:**
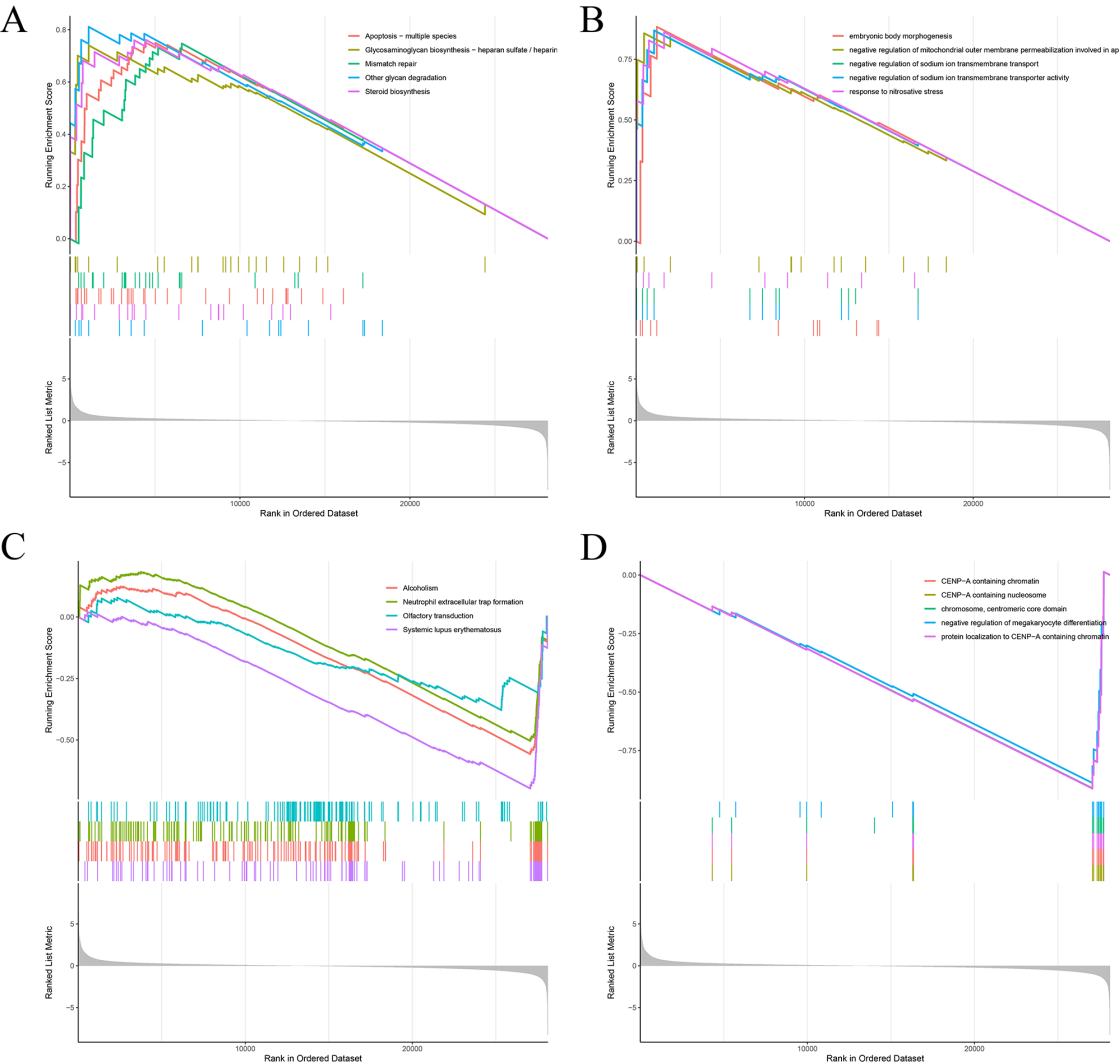
Gene Set Enrichment Analysis. (**A**) Top six up-regulated pathways in the GO dataset (sorted by NES). (**B**) Top six up-regulated pathways in the KEGG dataset. (**C**) Down six up-regulated pathways in the GO dataset. (**D**) Down six up-regulated pathways in the KEGG dataset

Pathway annotations for the remaining five genes are shown in Supplementary Fig. 1B. In our analysis, DDB1 emerged as an independent prognostic factor for BLCA based on multivariate Cox regression analysis. The initial GSEA using the C5-GO dataset (Fig. 9A) revealed that genes associated with DDB1 are enriched in pathways critical to apoptosis across multiple species, glycosaminoglycan biosynthesis (particularly heparan sulfate/heparin), mismatch repair, other glycan degradation, and steroid biosynthesis. Further investigation with the C2 KEGG dataset (Fig. 9B) uncovered the involvement of DDB1-related genes in processes such as embryonic body morphogenesis; negative regulation of mitochondrial outer membrane permeabilization; as well as the negative regulation of sodium ion transmembrane transport and transporter activity; and in mediating responses to nitrosative stress. Additional analysis using the C5-GO dataset (Fig. 9C) indicated that DDB1-associated genes are enriched in down-regulated pathways, including those linked to alcoholism, neutrophil extracellular trap formation, olfactory transduction, and systemic lupus erythematosus. Similarly, extending the analysis to the C2 KEGG dataset (Fig. 9D) demonstrated enrichment in down-regulated pathways involving CENP-A-containing chromatin and nucleosomes, the centromeric core domain, the negative regulation of megakaryocyte differentiation, and protein localization to CENP-A-containing chromatin. Taken together, these GSEA results highlight the significant role that DDB1 may play in regulating a spectrum of important biological processes associated with BLCA, and underscore its potential as a therapeutic target and biomarker for disease progression and prognosis.

## Discussion

BLCA is one of the most common malignancies of the genitourinary system, characterised by a complex aetiology involving multiple pathogenic mechanisms and diverse clinical manifestations - a reality that significantly hampers effective diagnosis and treatment [27]. Evidence indicates that bladder cancer represents a molecularly heterogeneous group of diseases, each following a distinct clinical trajectory and exhibiting varied responses to treatment [28]. The limited understanding of the interplay between BLCA molecular characteristics and clinical features has substantially impeded the development of personalized therapeutic strategies. Abnormal protein neddylation modifications have been increasingly associated with the progression of various tumors [12–14]. This post-translational modification plays a critical role in cell cycle progression, DNA repair, apoptosis, and the regulation of several tumor-related signaling pathways [12–14]. Moreover, dysregulation of neddylation activity shows a strong correlation with enhanced tumor growth, proliferation, and therapeutic resistance across multiple cancer types [29, 30]. Therefore, a deeper investigation into the mechanisms governing neddylation in BLCA and its impact on biological behaviour remains crucial to elucidate its role in disease progression and prognosis.

In BLCA, the neddylation-associated protein developmentally downregulated 8 (NEDD8) was found to be overexpressed in cancer tissues and correlated with reduced patient survival. Overexpression of NEDD8 was further shown to promote the proliferation, migration, and invasion of BLCA cells [31]. Differential gene analysis revealed 2974 genes with significantly altered expression, suggesting their potential roles in the onset and progression of BLCA. WGCNA subsequently organized these genes into 12 modules, identifying 1412 genes with significant prognostic relevance. An intersection analysis refined this collection to 32 neddylation-related genes strongly associated with BLCA. Ultimately, analysis led to the identification of six key neddylation-related genes—CUL1, PUM2, UBE2D3, HIF3A, COPS2, and DDB1—as potential prognostic factors. Furthermore, a prognostic model based on these critical variable genes was constructed, accurately predicting the survival outcomes of BLCA patients, with AUC values of 0.793, 0.792, and 0.773 at 1, 3, and 5 years, respectively.

The ubiquitinase DNA damage-specific binding protein 1 (DDB1) is classified as a DDB1-CUL4 related factor (DCAF) within the E3 ubiquitin ligase family [32]. DDB1 partners with DCAF to form a complex that selectively recognizes target protein substrates, catalyzing their ubiquitination and subsequent degradation [32, 33]. This mechanism influences tumor development through various pathways, including DNA damage repair, cell cycle regulation, apoptosis, invasion and metastasis, premature cellular senescence, proliferation, and the maintenance of tumor stem cell colonies [31–34]. In the present analysis, DDB1 emerged as an independent prognostic factor for BLCA based on multivariate Cox regression results. Complementary GO, KEGG, and GSEA analyses revealed that the DDB1 gene is highly correlated with apoptotic processes across species, mismatch repair, embryogenesis, negative regulation of sodium transmembrane transporter activity, and several tumor-activating signaling pathways. Notably, DDB1 exhibited statistically significant overexpression in tumor tissues, suggesting its pivotal role in modulating key biological processes and contributing to the complex progression of BLCA.

The other five genes are also highly enriched in bladder cancer. Through enrichment analysis, we found that CUL1 and UBE2D3 are mainly involved in immune-related pathways such as chemokine ligand-receptor interactions and the IL-17 signalling pathway. HIF3A and COPS2 are mainly associated with neurotransmitter signalling and receptor-related pathways, while PUM2 is enriched in bile acid metabolism pathways. In other studies The increased expression of UBE2D3 and PUM2 is associated with a better prognosis in patients with oesophageal cancer [35]. In contrast, in colorectal cancer, lower CUL1 expression correlates with better outcomes [36], while in breast cancer, reduced HIF3A levels indicate a more favorable prognosis [37]. Similarly, diminished expression of DDB1 in pancreatic cancer is linked to improved patient survival [38], and high levels of COPS2 have been shown to predict the occurrence of gastric cancer [39]. To further elucidate the relationship between the neddylation-related genes (including CUL1, PUM2, UBE2D3, HIF3A, COPS2, and DDB1) and BLCA prognosis, scRNA-seq was utilized to determine their cellular localization. The analysis revealed that UBE2D3 and COPS2 are highly expressed in seven different bladder cancer cell types, whereas HIF3A and CUL1 are expressed at low levels in the same cell clusters. In addition, DDB1 shows high expression in endothelial, epithelial, and smooth muscle cells, and PUM2 is predominantly expressed in endothelial cells. These differential expression patterns indicate the potential roles of these genes in oncogenesis, immunomodulation, and tumor-immune cell interactions. Collectively, these findings highlight the molecular complexity and clinical significance of BLCA and underscore the promise of neddylation-related genes as prognostic markers. Further investigation into the effects and mechanisms of neddylation in BLCA may provide novel targets for clinical management.

## Conclusion

CUL1, PUM2, UBE2D3, HIF3A, COPS2, and DDB1 serve as potential independent prognostic indicators for BLCA survival. A risk score model derived from these genes demonstrates robust accuracy in forecasting the survival outcomes of bladder cancer patients.

## Electronic supplementary material

Below is the link to the electronic supplementary material.

Supplementary Material 1: Supplementary Fig. 1. (A) The coorelation of hub genes. (B) KEGG enrichment of the hub genes.

## Data Availability

Analyses and visualisations of the scRNA-seq, RNA-seq and TCGA datasets from this study are available in the GEO database. Other data from this study are available from the corresponding author.

## References

[1] Compérat E, Amin MB, Cathomas R, Choudhury A, De Santis M, Kamat A, Stenzl A, Thoeny HC, Witjes JA. Current best practice for bladder cancer: a narrative review of diagnostics and treatments.lancet. 2022;400(10364):1712–21. 10.1016/S0140-6736(22)01188-6 36174585

[2] Lenis AT, Lec PM, Chamie K, Mshs MD. Bladder Cancer: Rev JAMA. 2020;324(19):1980–91. 10.1001/jama.2020.17598 33201207

[3] Dobruch J, Daneshmand S, Fisch M, Lotan Y, Noon AP, Resnick MJ, Shariat SF, Zlotta AR, Boorjian SA. Gender and bladder cancer: A collaborative review of etiology, biology, and outcomes. Eur Urol. 2016;69(2):300–10. 26346676 10.1016/j.eururo.2015.08.037

[4] Lobo N, Afferi L, Moschini M, Mostafid H, Porten S, Psutka SP, Gupta S, Smith AB, Williams SB, Lotan Y, Epidemiology. Screening, and prevention of bladder Cancer. Eur Urol Oncol. 2022;5(6):628–39. 36333236 10.1016/j.euo.2022.10.003

[5] Ahmadi H, Duddalwar V, Daneshmand S. Diagnosis and staging of bladder Cancer. Hematol Oncol Clin North Am. 2021;35(3):531–41. 33958149 10.1016/j.hoc.2021.02.004

[6] Alfred Witjes J, Max Bruins H, Carrión A, Cathomas R, Compérat E, Efstathiou JA, Fietkau R, Gakis G, Lorch A, Martini A, Mertens LS, Meijer RP, Milowsky MI, Neuzillet Y, Panebianco V, Redlef J, Rink M, Rouanne M, Thalmann GN, Sæbjørnsen S, Veskimäe E, van der Heijden AG. European association of urology guidelines on Muscle-invasive and metastatic bladder cancer: summary of the 2023 guidelines. Eur Urol. 2024;85(1):17–31. 37858453 10.1016/j.eururo.2023.08.016

[7] Teoh JY, Kamat AM, Black PC, Grivas P, Shariat SF, Babjuk M. Recurrence mechanisms of non-muscle-invasive bladder cancer - a clinical perspective. Nat Rev Urol. 2022;19(5):280–94. 35361927 10.1038/s41585-022-00578-1

[8] Lai H, Cheng X, Liu Q, Luo W, Liu M, Zhang M, Miao J, Ji Z, Lin GN, Song W, Zhang L, Bo J, Yang G, Wang J, Gao WQ. Single-cell RNA sequencing reveals the epithelial cell heterogeneity and invasive subpopulation in human bladder cancer. Int J Cancer. 2021;149(12):2099–115. 34480339 10.1002/ijc.33794

[9] Shi ZD, Sun Z, Zhu ZB, Liu X, Chen JZ, Hao L, Zhu JF, Pang K, Wu D, Dong Y, Liu YF, Chen WH, Liang Q, Zhuo SC, Han CH. Integrated single-cell and Spatial transcriptomic profiling reveals higher intratumour heterogeneity and epithelial-fibroblast interactions in recurrent bladder cancer. Clin Transl Med. 2023;13(7):e1338. 37488671 10.1002/ctm2.1338 PMC10366350

[10] Cui Y, Chen Z, Pan B, Chen T, Ding H, Li Q, Wan L, Luo G, Sun L, Ding C, Yang J, Tong X, Zhao J. Neddylation pattern indicates tumor microenvironment characterization and predicts prognosis in lung adenocarcinoma. Front Cell Dev Biol. 2022;10:979262. 36176276 10.3389/fcell.2022.979262 PMC9513323

[11] Liu H, Shih YH, Wang WL, Chang WL, Wang YC. UBE1C is upregulated and promotes neddylation of p53 in lung cancer. FASEB J. 2023;37(10):e23181. 37668436 10.1096/fj.202300629R

[12] Zhou L, Jiang Y, Luo Q, Li L, Jia L. Neddylation: a novel modulator of the tumor microenvironment. Mol Cancer. 2019;18(1):77. 30943988 10.1186/s12943-019-0979-1 PMC6446326

[13] Lu Y, Yang X. The pivotal roles of neddylation pathway in immunoregulation. Immun Inflamm Dis. 2020;8(4):782–92. 32749072 10.1002/iid3.335 PMC7654410

[14] Zhu J, Chu F, Zhang M, Sun W, Zhou F. Association between neddylation and immune response. Front Cell Dev Biol. 2022;10:890121. 35602593 10.3389/fcell.2022.890121 PMC9117624

[15] Zhou Q, Lin W, Wang C, Sun F, Ju S, Chen Q, Wang Y, Chen Y, Li H, Wang L, Hu Z, Jin H, Wang X, Sun Y. Neddylation Inhibition induces glutamine uptake and metabolism by targeting CRL3(SPOP) E3 ligase in cancer cells. Nat Commun. 2022;13(1):3034. 35641493 10.1038/s41467-022-30559-2 PMC9156729

[16] Ritchie ME, Phipson B, Wu D, Hu Y, Law CW, Shi W, Smyth GK. Limma powers differential expression analyses for RNA-sequencing and microarray studies. Nucleic Acids Res. 2015;43(7):e47. 25605792 10.1093/nar/gkv007 PMC4402510

[17] Lu J, Zhang Y, Wang S, Bi Y, Huang T, Luo X, Cai YD. Analysis of four types of leukemia using gene ontology term and Kyoto encyclopedia of genes and genomes pathway enrichment scores. Comb Chem High Throughput Screen. 2020;23(4):295–303. 30599106 10.2174/1386207322666181231151900

[18] Langfelder P, Horvath S. WGCNA: an R package for weighted correlation network analysis. BMC Bioinformatics. 2008;9:559. 19114008 10.1186/1471-2105-9-559 PMC2631488

[19] Szklarczyk D, Gable AL, Lyon D, Junge A, Wyder S, Huerta-Cepas J, Simonovic M, Doncheva NT, Morris JH, Bork P, Jensen LJ, Mering CV. STRING v11: protein-protein association networks with increased coverage, supporting functional discovery in genome-wide experimental datasets. Nucleic Acids Res. 2019;47(D1):D607–13. 30476243 10.1093/nar/gky1131 PMC6323986

[20] Shannon P, Markiel A, Ozier O, Baliga NS, Wang JT, Ramage D, Amin N, Schwikowski B, Ideker T. Cytoscape: a software environment for integrated models of biomolecular interaction networks. Genome Res. 2003;13(11):2498–504. 14597658 10.1101/gr.1239303 PMC403769

[21] Wang H, Lengerich BJ, Aragam B, Xing EP. Precision Lasso: accounting for correlations and linear dependencies in high-dimensional genomic data. Bioinformatics. 2019;35(7):1181–7. 30184048 10.1093/bioinformatics/bty750 PMC6449749

[22] Wang Q, Qiao W, Zhang H, Liu B, Li J, Zang C, Mei T, Zheng J, Zhang Y. Nomogram established on account of Lasso-Cox regression for predicting recurrence in patients with early-stage hepatocellular carcinoma. Front Immunol. 2022;13:1019638. 36505501 10.3389/fimmu.2022.1019638 PMC9726717

[23] Iasonos A, Schrag D, Raj GV, Panageas KS. How to build and interpret a nomogram for cancer prognosis. J Clin Oncol. 2008;26(8):1364–70. 18323559 10.1200/JCO.2007.12.9791

[24] George B, Seals S, Aban I. Survival analysis and regression models. J Nucl Cardiol. 2014;21(4):686–94. 24810431 10.1007/s12350-014-9908-2 PMC4111957

[25] Zhang Y, Jiang S, He F, Tian Y, Hu H, Gao L, Zhang L, Chen A, Hu Y, Fan L, Yang C, Zhou B, Liu D, Zhou Z, Su Y, Qin L, Wang Y, He H, Lu J, Xiao P, Hu S, Wang QF. Single-cell transcriptomics reveals multiple chemoresistant properties in leukemic stem and progenitor cells in pediatric AML. Genome Biol. 2023;24(1):199. 37653425 10.1186/s13059-023-03031-7 PMC10472599

[26] Chen H, Yang W, Li Y, Ma L, Ji Z. Leveraging a disulfidptosis-based signature to improve the survival and drug sensitivity of bladder cancer patients. Front Immunol. 2023;14:1198878. 37325625 10.3389/fimmu.2023.1198878 PMC10266281

[27] Ward Grados DF, Ahmadi H, Griffith TS, Warlick CA. Immunotherapy for bladder cancer: latest advances and ongoing clinical trials. Immunol Invest. 2022;51(8):2226–51. 36083246 10.1080/08820139.2022.2118606

[28] Kamoun A, de Reyniès A, Allory Y, Sjödahl G, Robertson AG, Seiler R, Hoadley KA, Groeneveld CS, Al-Ahmadie H, Choi W, Castro MAA, Fontugne J, Eriksson P, Mo Q, Kardos J, Zlotta A, Hartmann A, Dinney CP, Bellmunt J, Powles T, Malats N, Chan KS, Kim WY, McConkey DJ, Black PC, Dyrskjøt L, Höglund M, Lerner SP, Real FX, Radvanyi F. Bladder Cancer molecular taxonomy group.a consensus molecular classification of Muscle-invasive bladder Cancer. Eur Urol. 2020;77(4):420–33. 31563503 10.1016/j.eururo.2019.09.006 PMC7690647

[29] He ZX, Yang WG, Zengyangzong D, Gao G, Zhang Q, Liu HM, Zhao W. Ma LY.Targeting Cullin neddylation for cancer and fibrotic diseases. Theranostics. 2023;13(14):5017–56. 37771770 10.7150/thno.78876 PMC10526667

[30] Bano I, Malhi M, Zhao M, Giurgiulescu L, Sajjad H, Kieliszek M. A review on Cullin neddylation and strategies to identify its inhibitors for cancer therapy. 3 Biotech. 2022;12(4):103. 35463041 10.1007/s13205-022-03162-x PMC8964847

[31] Tian DW, Wu ZL, Jiang LM, Gao J, Wu CL, Hu HL. Neural precursor cell expressed, developmentally downregulated 8 promotes tumor progression and predicts poor prognosis of patients with bladder cancer. Cancer Sci. 2019;110(1):458–67. 30407690 10.1111/cas.13865 PMC6317957

[32] Beecher M, Kumar N, Jang S, Rapić-Otrin V, Van Houten B. Expanding molecular roles of UV-DDB: shining light on genome stability and cancer. DNA Repair (Amst). 2020;94:102860. 32739133 10.1016/j.dnarep.2020.102860 PMC7873659

[33] Sang Y, Yan F, Ren X. The role and mechanism of CRL4 E3 ubiquitin ligase in cancer and its potential therapy implications. Oncotarget. 2015;6(40):42590–602. 26460955 10.18632/oncotarget.6052 PMC4767455

[34] Wittschieben BØ, Wood RD. DDB complexities. DNA Repair (Amst). 2003;2(9):1065–9. 12967661 10.1016/s1568-7864(03)00113-7

[35] Guan GG, Wang WB, Lei BX, Wang QL, Wu L, Fu ZM, Zhou FX, Zhou YF. UBE2D3 is a positive prognostic factor and is negatively correlated with hTERT expression in esophageal cancer. Oncol Lett. 2015;9(4):1567–74. 25789002 10.3892/ol.2015.2926 PMC4356423

[36] Wang W, Deng J, Wang Q, Yao Q, Chen W, Tan Y, Ge Z, Zhou J, Zhou Y. Synergistic role of Cul1 and c-Myc: prognostic and predictive biomarkers in colorectal cancer. Oncol Rep. 2017;38(1):245–52. 28560438 10.3892/or.2017.5671

[37] Shen J, Song R, Ye Y, Wu X, Chow WH, Zhao H. HIF3A DNA methylation, obesity and weight gain, and breast cancer risk among Mexican American women. Obes Res Clin Pract. 2020 Nov-Dec;14(6):548–53. 10.1016/j.orcp.2020.10.001 33121895

[38] Zhang Y, Lei Y, Xu J, Hua J, Zhang B, Liu J, Liang C, Meng Q, Yu X, Shi S. Role of damage DNA-Binding protein 1 in pancreatic Cancer progression and chemoresistance. Cancers (Basel). 2019;11(12):1998. 31842285 10.3390/cancers11121998 PMC6966444

[39] Yang L, Wang J, Li J, Zhang H, Guo S, Yan M, Zhu Z, Lan B, Ding Y, Xu M, Li W, Gu X, Qi C, Zhu H, Shao Z, Liu B, Tao SC. Identification of serum biomarkers for gastric Cancer diagnosis using a human proteome microarray. Mol Cell Proteom. 2016;15(2):614–23. 10.1074/mcp.M115.051250 PMC4739676 26598640

