# Supplementary figures and images for: Prognostic analysis of bladder cancer with neddylation-related genes
Source: Hereditas. 2025 Jun 16;162:105. doi: 10.1186/s41065-025-00463-y (PMC12172356; doi:10.1186/s41065-025-00463-y)

**Supplementary Fig. 1. (A) The coorelation of hub genes. (B) KEGG enrichment of the hub genes.**


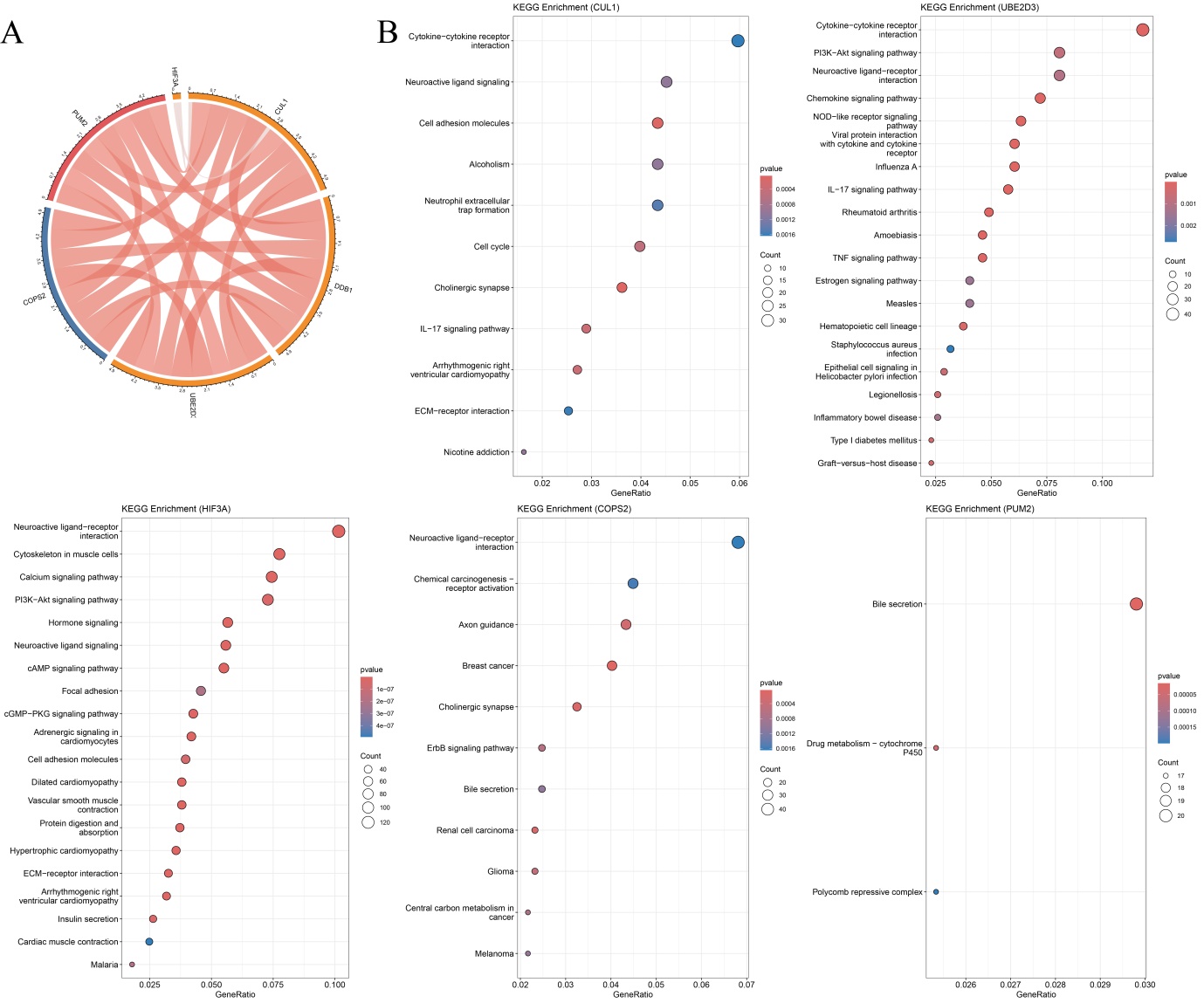

Supplement: Supplementary file 1 — Supplementary Material 1: Supplementary Fig. 1. (A) The coorelation of hub genes. (B) KEGG enrichment of the hub genes. [file 41065_2025_463_MOESM1_ESM.docx]
